# National survey of the Portuguese elderly nutritional status: study protocol

**DOI:** 10.1186/s12877-016-0299-x

**Published:** 2016-07-16

**Authors:** Teresa Madeira, Catarina Peixoto-Plácido, Beatriz Goulão, Nuno Mendonça, Violeta Alarcão, Nuno Santos, Rita Machado de Oliveira, Agneta Yngve, Asta Bye, Astrid Bergland, Carla Lopes, Paulo Nicola, Osvaldo Santos, João Gorjão Clara

**Affiliations:** Instituto de Medicina Preventiva e Saúde Pública da Faculdade de Medicina, Universidade de Lisboa, Av. Prof. Egas Moniz, 1649-028 Lisboa, Portugal; Instituto de Saúde Ambiental da Faculdade de Medicina, Universidade de Lisboa, Av. Prof. Egas Moniz, 1649-028 Lisboa, Portugal; Faculdade de Medicina, Universidade de Lisboa, Av. Prof. Egas Moniz, 1649-028 Lisboa, Portugal; Health Services Research Unit, University of Aberdeen, Foresterhill, Aberdeen, AB25 2ZD UK; Institute for Ageing, Newcastle University, Newcastle-upon-tyne, NE1 7RU UK; Department of Food, Nutrition and Dietetics, Uppsala University, P.O. Box 256, SE 75 105, Uppsala, Sweden; Oslo and Akershus University College of Applied Sciences, Pilestredet 46, 0167 Oslo, Norway; Regional Advisory Unit for Palliative Care, Oslo University Hospital, Box 4965, Nydalen, 0424 Oslo, Norway; EPIUnit–Institute of Public Health, University of Porto, Rua das Taipas 135, 4050-600 Porto, Portugal; Faculty of Medicine, University of Porto, Al. Prof. Hernâni Monteiro, 4200-319 Porto, Portugal

**Keywords:** Nutritional status, Undernutrition, Malnutrition, Nutritional assessment, Ageing, Elderly, National survey, Epidemiological study

## Abstract

**Background:**

Worldwide we are facing a serious demographic challenge due to the dramatic growth of the population over 60 years. It is expected that the proportion of this population will nearly double from 12 to 22 %, between 2015 and 2050. This demographic shift comes with major health and socio-economic concerns. Nutrition is a fundamental determinant of both health and disease and its role in extending a healthy lifespan is the object of considerable research. Notably, malnutrition is one of the main threats to health and quality of life among the elderly. Therefore, knowledge about nutritional status among the elderly is essential for the promotion and maintenance of healthy ageing and to support the development of health protection policies and equity in elderly health care.

**Methods:**

This is a nationwide nutrition survey of the Portuguese population over 65 years old, with data collection through face-to-face interviews. A representative and random sample of community dwelling elderly and nursing homes residents will be obtained by multistage sampling stratified per main Portuguese regions, sex and age groups. Minimum sample size was estimated to be 2077 elderly (979 in the community and 1098 in nursing homes). Data will be collected on food habits and eating patterns, nutritional status, food insecurity, lifestyle, self-rated general health status and self-reported diseases, functionality, loneliness, cognitive function, emotional status and demographic and socio-economic characterization.

**Discussion:**

This is the first national survey to evaluate the prevalence of nutritional risk and malnutrition of the Portuguese population above 65 years old, including those living in nursing homes. It will allow the identification of population subgroups of elderly with increased odds of malnutrition and nutritional risk. In addition, this survey will contribute to the identification of psychosocial and clinical predictors of malnutrition among elderly, which is an important risk factor for other devastating medical conditions.

## Background

The increased life expectancy in economically developing and developed countries is reflected in the growth of the population over 65 years old. In the European Union, data from 2014, shows an increase of 0.3 % of persons aged 65 and over compared with the previous year, representing a 18.5 % share of the total population [1]. In 2014, 20.3 % of the Portuguese population was over 65 years old; therefore Portugal is the fourth country within the European Union with more elderly people [2].

Over the last 20 years, life expectancy has increased by six years. Unfortunately, increased life expectancy does not necessarily mean increased quality of life. As a matter of fact, it is hypothesized that aging-associated diseases are solely postponed in time leading to a higher prevalence of poor health in the oldest old [3]. Moreover, this demographic shift poses major challenges to the healthcare system and societies [4, 5].

In the elderly population, adequate diet and nutritional status are important health determinants [6, 7]. Noteworthy, adequate nutrition can either prevent, delay or significantly improve a large proportion of chronic diseases affecting older adults [8–10]. Malnutrition in older people is a common problem that brings many negative outcomes, such as decreased quality of life, medical complications, hospitalization and even higher mortality [11, 12]. Promoting a healthy diet has therefore the potential to substantially reduce the burden of disease and to improve quality of life. Overall, nutrition intervention among the elderly encloses the potential to promote healthier and more active ageing.

Malnutrition is as a state in which a deficiency, excess or imbalance of energy, protein or other nutrients causes adverse effects on function and clinical outcome. Therefore, malnutrition can either refer to overnutrition, undernutrition or to an unbalanced diet [13, 14]. In the literature the term is most often used to describe undernutrition. Given the increased life expectancy and the fact that malnutrition is frequent, especially among the elderly population, it is expected that the number of older people who are malnourished or at risk of developing malnutrition will increase. However, malnutrition is not solely determined by the ageing process. Several other factors such as low levels of education, poor financial status, chronic diseases, social isolation and reduced physical functional capacity play an important role [15–20].

The prevalence of malnutrition among the elderly population demands the urgent development of novel and more effective approaches capable of performing an early diagnosis and efficient treatment, thus promoting a meaningful life, an increased and healthier lifespan [21]. Malnutrition is often subtle in older adults and its diagnosis requires specific screening tools and health professionals’ awareness and adequate training [22]. Therefore, there is a widespread demand for adequate nutritional screening in high-risk populations and environments. Thus, good instruments for assessing nutritional status and dietary intake are essential for the design of effective interventions, guidelines and policies. In order to develop adequate and personalized nutritional care plans, healthcare organizations should have clear policies and simple protocols to identify patients at nutritional risk [23].

It is difficult to make universal recommendations about nutritional screening and intervention plans because the prevalence and types of nutritional problems vary according to country, health care setting and local resources [24]. Thus, it is of utmost importance to develop specific tools to collect national data on this important topic. The promotion of good nutrition in the elderly, together with early diagnosis and treatment of malnutrition could improve health and minimize significantly the associated-social and economic burden. Having a more comprehensive view of this problem, including prevalence, identifying possible predictors and groups at risk will allow health professionals and elderly care givers to better tackle malnutrition and plan customized interventions. In addition, such approach will enable governments to identify and take the appropriate action in terms of public health. All together this prompted us to develop the present protocol. Moreover, in Portugal there is no updated data about the elderly nutritional status and dietary habits. The first and only national food and nutrition survey was conducted about 35 years ago, and only now a new National Dietary Survey is being conducted, with which the current project shares the community sampling and data collection procedures [25]. More than that, there was never a survey targeted to the national elderly population, covering the community and nursing homes and without an upper age limit.

Despite the lack of data regarding the Portuguese reality, there are large international multi-center studies that alert to the fact that malnutrition is a widespread problem in older adults (in community dwelling older adults: 4.2 % are malnourished and 27.4 % are at risk of malnutrition) [26]. Moreover, in nursing homes, this prevalence is reported to raise to 52.1 % of elderly at risk of malnutrition and 27.2 % of malnourished [27]. These high prevalence figures are intertwined with serious health complications, compromising quality of life and leading to substantial costs for health care systems and society in general [28, 29].

The present protocol is part of a larger project, the PEN-3S: Portuguese Elderly Nutritional Status Surveillance System, aiming at the elderly’s health promotion and protection. The PEN-3S includes two research components: 1) assessing the nutritional status and dietary habits of the Portuguese resident population aged 65 and over (individuals from 65 to 84 years of age will be sampled as part of the National Food, Nutrition and Physical Activity Survey: IAN-AF [25]); 2) developing and testing an electronic nutritional status surveillance system both at primary care level and at the nursing homes. This nutritional status surveillance system is still under development and will be described elsewhere.

The present protocol aims at characterizing the nutritional status of the Portuguese elderly population. The data set collected by this survey will support the development of health protection policies and health care equity. The proposed study has two major goals: (i) to characterize the nutritional status of the Portuguese population aged 65 and over, by sex, age groups and regions, in the community and in nursing homes, (ii) to identify and characterize predictors of malnutrition among the Portuguese elderly by sex, age and region, in the community and in nursing homes.

## Methods

### Design

This is a nationwide cross-sectional observational study of the Portuguese elderly population aged 65 and over.

### Sample

The study will include a representative sample, at national level, of individuals aged 65 and over, living in the community and in nursing homes across Portugal (mainland and Açores and Madeira islands).

**Fig. 1:**
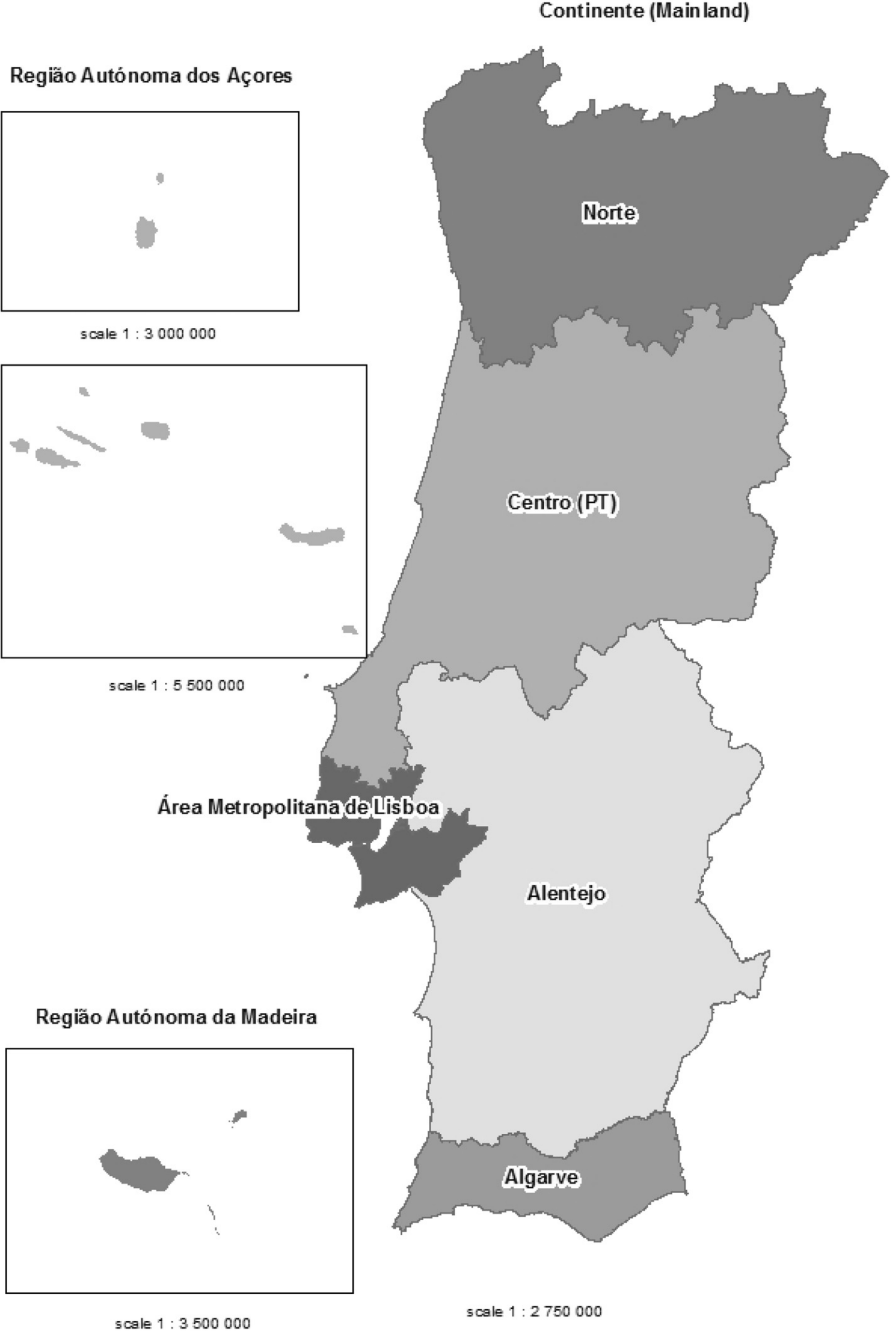
Map illustrating the seven official territorial division NUTS II regions where data will be collected

For the nursing homes, a cluster random sampling will be performed in each of the seven basic regions for the application of regional policies: NUTS II (nomenclature of territorial units for statistics, as defined by the European Union (Fig. 1).

The clusters are composed by nursing homes registered with the Portuguese Social Security Institute covering all the national nursing homes, either private or supported by the government. In each region, nursing homes will be randomly (by quota) selected, ensuring a minimum of 6 residential homes per region. All the elderly who meet the inclusion criteria and do not meet the exclusion criteria, living in each selected nursing home, will be invited to participate (census procedure).

**Fig. 2:**
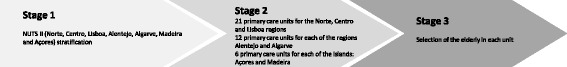
Phases of random sampling

The community sample will be selected by a multistage approach, according to the methodology defined by the IAN-AF and using the following steps (Fig. 2):

1. NUTS II stratification (Norte, Centro, Área Metropolitana de Lisboa, Alentejo, Algarve, Região Autónoma da Madeira e Região Autónoma dos Açores);
2. selection of 21 primary care units for each Norte, Centro and Lisboa regions, 12 primary care units for each of the regions Alentejo and Algarve, and 6 primary care units for each of the regions Açores and Madeira. In the selected primary healthcare care units, participants will be randomly selected from the population registered and not necessarily user of the National Health System (99 % of the Portuguese population is registered in the national Health Care System [30]).
3. random selection of the elderly registered in each unit.

We expect to collect data from more than 2000 individuals over 65 years, living in the community and in nursing homes across Portugal. This number is based on the 27 % estimated prevalence for nutritional risk in the community and 67 % for residents in nursing homes. Taking into account 3 % of overall error, we calculated the sample size of 2077 individuals aged 65 and over (979 in the community and of 1098 in nursing homes). In order to calculate sample size per age and sex group, we took into consideration the Portuguese population distribution (based on the numbers from the Statistics Portugal, 2011). The 85 and over age group will be over-sampled, thus guaranteeing a maximum error of 10 % per strata.

In the community sample, accounting for 50 % of response rate, due to non-contactable individuals, incomplete questionnaires, non-responses, 1470 individuals will be selected and invited to participate in the study. Selected individuals that refuse to participate will be characterized on basis of the available socio-demographic information.

**Fig. 3:**
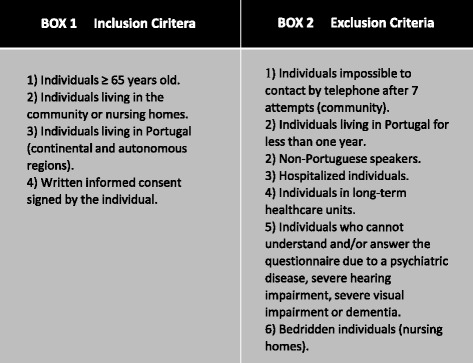
Exclusion and Inclusion Criteria

Any individual aged 65 and over who meet the inclusion and do not meet the exclusion criteria are eligible (Fig. 3).

Selected individuals will be personally invited to enroll in the study. This invitation will be done by phone (primary care units) or by face-to-face contact (nursing homes). In the first contact, a brief presentation of the study and research team will be carried out. For the elderly living in residential homes will be invited by the interviewers to participate in the study. Should the individual accept to enroll in the study, an appointment will be made to his/her convenience in order to conduct the interview, either at his/her primary healthcare unit or at home (for those participants with accessibility difficulties).

### Data collection

Data collection will be based on a computer-assisted face-to-face structured interview followed by anthropometric measurements, conducted by formally trained professionals (nutritionists and dietitians). These professionals received specific training regarding interviewing techniques with the elderly and collection of anthropometric measures. Data collection will be carried out in a maximum of 12 months.

The interview will collect data regarding (a) demographic and socioeconomic characterization, including age, sex, nationality, education level, marital status, household size, employment status, monthly income, (b) self-perception of general health status and self-reported morbidity (cardiac disease, stroke, cancer, diabetes mellitus I or II, hypertension, dyslipidemia, gastrointestinal disease, arthrosis), (c) lifestyles (including physical activity and smoking habits), dietary habits and food intake, (d) food insecurity, (e) nutritional status, (f) cognitive function, (g) emotional status, (h) loneliness perception and (i) functionality. Variables from a) to d) share the methodology with the IAN-AF, and are integrated in a specific electronic platform “YOU, eAT& MOVE” developed by the IAN-AF [25]. Table 1 provides an overview of the validated instruments included in the interview.

**Table 1:** Instruments used in the survey (computer-assisted face-to-face data collection)

Assessed Constructs	Questionnaire/measure scale	Description
Health status self-perception	General self-rated health status	Single-item scale, with a five-point scale of answer, from 1 “Poor” to 5 “Excellent” health. This question is extensively used, and is a key predictor of severe morbidity and even mortality [32]. It is also often used as a component of the health-related quality of life, included in the Medical Outcome Study Short Form 36 (MOS SF-36) scale. Its psychometric properties were described for Portugal [33].
Physical activity	IPAQ: International Physical Activity Questionnaire, short form	This is a 9-items scale, providing information on the time spent walking, in vigorous and moderate intensity activities and in sedentary activities. It is validated for the Portuguese population [34, 35]. Additional questions regarding number of sleeping hours, physical activity associated with daily tasks, identification of the leisure and programmed physical activity were added.
Food consumption and food patterns	24 h recall	Dietary assessment will be performed by using a 24 h recall interview. In this interview, respondents are inquired about the food consumed in the previous 24 h, in two random non-consecutive days, without prior notification, within a timeframe between 8 and 15 days. Portion size estimation will be performed by using food pictures and standardized household measures integrated in the software “YOU, eAT&Move”. The software automatically calculates total nutrient intake. All the methodology was developed in the scope of IAN-AF, following the methodology proposed by the European Food Safety Authority (EFSA) in the pan-European Survey EU-MENU [36]. A Food Propensity Questionnaire (FPQ) will be used to complement the 24 h recall. The FPQ includes a list of 79 food items (including alcoholic beverages), consumed in the last 12 months. For each item, there is a frequency-based ordinal scale, with nine possible responses ranging from “never” to “6-7 days/week”. The FPQ was adapted by the IAN-AF team, following the protocol proposed by the Food Consumption Data Collection Methodology for the EU Menu Survey (Pilot-PANEU) [36, 37].
	Food Propensity Questionnaire	
Food insecurity		Food insecurity as a measure of hunger due to income limitations represents the condition of the household members as a group. This is a continuous, linear scale [38–40].
Anthropometric measurements	Weight Height Waist, hip, arm and calf circumferences.	Individuals wearing minimal clothing without shoes will be measured to the nearest 0.1 kg with a portable calibrated scale (SECA Robusta 813®). Height will be measured without shoes using a portable stadiometer to the nearest 0.1 cm (SECA 214®). Whenever collection of height using the stadiometer is not possible, estimation of height will be done through hand length. This will be measured using a fiberglass tape to the nearest 0.1 cm and a validated equation for the Portuguese population [41]. Waist, hip, arm and calf circumferences to the nearest 0.1 cm will be measured with a non-extensible, flexible, fiberglass tape (SECA 201®). All measurements will be performed according to a standard interviewer manual based on the International Standards for Anthropometric Assessment (ISAK) [42].
Nutritional status	MNA®: Mini Nutritional Assessment	This is the most widely used and validated screening method for identification of frail elderly and geriatric population at nutritional risk. It is recommended by different national and international clinical and scientific organizations as a community useful clinical tool. It is composed by 18-items, giving a maximum score of 30 points. The cut-off of below 24 points is used to identify individuals at-risk and to predict poor outcomes in the elderly [43–45]. This instrument is validated for the Portuguese population [46].
Cognitive function	MMSE: Mini-Mental State Examination	This is one of the most widely used instruments in epidemiological studies, as a screening of cognitive impairment. It includes 30 items, assessing temporal and spacial orientation, working memory, recall, attention, arithmetic capacity, linguistic, and visual-motor skills. The maximum score is 30 points (one point per correct item). The minimum cut-off for adequate cognitive functioning is set accordingly to the level of education of the participant [47, 48]. The psychometric properties of the MMSE for the Portuguese population were previously described [49].
Emotional status	GDS: Geriatric Depression Scale, Short Form	This is a 15-items instrument to screen for clinical depression among elderly. It excludes somatic symptoms that might be due to medical illness, and makes use of a simple response format: yes/no. The sum of scores allows the categorization of respondents in terms of depressed or non-depressed. The development, validation and factor structure of the shorter GDS-15 was described and evaluated in nursing home populations [50, 51]. The psychometric properties of the MMSE for the Portuguese population were previously described [52].
Loneliness	UCLA Loneliness Scale	This is a 16-items scale, with a 4-points Likert-type answer of format (from 1 “never” to 4 “frequently”) which measures loneliness. Scores range from 16 to 64 points (the highest the value the highest the subjective feeling of loneliness or social isolation) [53, 54]. The psychometric properties of the UCLA Loneliness Scale for the Portuguese population were previously described [55].
Functionality	Lawton Scale	This scale measures the instrumental daily living activities of the elderly. It is an 8-itens scale, with a polycotomic format of response, allowing the evaluation of the elderly autonomy to conduct daily life activities. Scores range from 0 to 8 [56]. The psychometric properties of the Lawton Scale for the Portuguese population were previously described [57].

Some general and descriptive data about the nursing homes will be collected, namely: type (public or government supported), number of residents, number of staff members who are assigned to support the elderly during meals, presence of a nutritionist and information regarding the meals (in-house cooking or catering service). Importantly, we will ask the nursing homes whether they screen for malnutrition and have a define intervention plan. This will be done through a short questionnaire addressed to the direction board of each participating nursing home.

### Statistical analysis

Statistical package IMB/SPSS® version 23 and R software (The R Project for Statistical Computing), version 3.2.3 will be used to perform data analysis. Assumed significance level for statistical inference is 5 %. Data will be analyzed to obtain descriptive statistics through complex sample analysis IBM/SPSS procedure. The normality of the distributions will be assessed through Kolmogorov-Smirnov test and Kurtosis and Skewness values. Continuous variables will be presented as mean values and standard deviation, while categorical and non-normally distributed variables will be reported as median and interquartile range. Differences between groups will be assessed using independent samples t-tests or Mann–Whitney *U* test (for continuous variables), chi-square test (for categorical variables), and ANOVA or Kruskal-Wallis (for more than 2 groups). Univariate and multivariable regressions (linear and logistic) analysis will be used to evaluate the associations between nutritional status (dependent variable) and the different variables in study in order to assess malnutrition predictors. Covariates with a p-value ≤0.10 and with some explanatory power will be included in one multivariate model. Food and nutritional patterns will be compared by sex and NUTS II region, after sampling design effect adjustment, considering an exposition-dependent effect according to community (primary care) or nursing home setting. Nutritional status estimates of usual consumption and inter and intra-individual variability assessed by 24 h recalls will be estimated through mixed effects models (random and fixed) using the method proposed by the National Research Council and the Institute of Medicine, US [31].

## Discussion

In the past, malnutrition in the elderly was often minimized or even neglected. With the dramatic increase of this subgroup of the population, there is a need for further research in the area of elderly nutritional status. Moreover, prevalence data are still lacking in many countries, including Portugal, and prevention and treatment of such serious condition and associated-diseases does not currently receive the expected attention. Thus, the protocol developed by the PEN-3S represents a relevant scientific contribute by providing the first characterization of the nutritional situation among the older people in Portugal and associated clinical psychosocial variables. These data will be of use by international agencies (e.g., WHO, Eurostat, OECD) for providing statistical elements that, ultimately, inform national social security and health policies. The results of the research here proposed will allow the identification and description of specific elderly subgroups that may be more susceptible to malnutrition. It will also pinpoint malnutrition predictors. These data will also be used to develop an electronic malnutrition surveillance system for the elderly, including screening, alert and referral components. This system is meant to be used in the primary health care facilities and in nursing homes. Overall, this study aims to shed light and increase awareness for the importance of nutritional screening among older people, with inherent implications for policy makers.

## Abbreviations

IAN-AF, National Food, Nutrition and Physical Activity Survey; NUTS II, Nomenclature of territorial units for statistics; PEN-3S, Portuguese Elderly Nutritional Status Surveillance System
